# Evaluation of Immune Dysregulation in Sepsis with a Composite Marker Gene Panel

**DOI:** 10.3390/biomedicines14030617

**Published:** 2026-03-10

**Authors:** Hongxing Lei

**Affiliations:** 1 China National Center for Bioinformation, Beijing 100101, China; leihx@big.ac.cn; Tel.: +86-010-84097276; 2 Beijing Institute of Genomics, Chinese Academy of Sciences, Beijing 100101, China; 3 Medical School, University of Chinese Academy of Sciences, Beijing 100408, China

**Keywords:** sepsis, SOFA, host response, neutrophil degranulation, interferon signaling, MHC class II

## Abstract

**Background**: Dysregulated host response to infection plays vital roles in the high mortality rate of sepsis. Better understanding of the dysregulated host response is required for the improved management of sepsis. **Methods**: In this work, we conducted side-by-side comparison of several components of host immunity in sepsis with a composite marker gene panel. This gene panel consisted of five sets of marker genes, including the NDrG gene set for neutrophil degranulation-related genes, the ISGa gene set for type I interferon signaling, the GBPs gene set for type II interferon signaling, the HLAd gene set for major histocompatibility (MHC) class II, and the LYMd gene set for lymphocytes and dendritic cells. Seven relevant transcriptome datasets were used for the evaluation. **Results**: It was found that sepsis was characterized by simultaneous activation of the NDrG gene set and suppression of the HLAd and LYMd gene sets. In contrast, both activation and suppression of the ISGa and GBPs gene sets were observed in sepsis. Compared to sepsis patients with hypo-inflammation, those with hyper-inflammation displayed higher values of NDrG and lower values of HLAd and LYMd. Reversal of gene dysregulation was observed in the NDrG, HLAd and LYMd gene sets for sepsis patients after treatment at intensive care unit (ICU), especially for those who responded better to the treatment. In a cohort of sepsis patients admitted to the ICU, the initial expression values of the NDrG, HLAd and LYMd gene sets were associated with the outcome. Notably, all patients with (NDrG-HLAd) < 1.5 survived, accounting for 75% of the survivors (45 out of 60). A simple combination of the (NDrG-HLAd) value and the sequential organ failure assessment (SOFA) score could identify 90% of the survivors with 3.5% false positive rate. **Conclusions**: Overall, this composite gene panel is applicable to the serial monitoring of immune dysregulation in sepsis. It also suggests that the NDrG and HLAd gene sets may be promising targets for immune modulation in sepsis.

## 1. Introduction

The hallmarks of sepsis are multi-organ damage and dysregulated host response to infection [1]. Multi-organ damage encompasses respiratory failure, cardiovascular dysfunction, acute kidney injury, liver injury, blood clotting, and brain dysfunction. This could be caused by dysregulated host response which includes both systemic hyper-inflammation and immunosuppression. Clinically, the SOFA score has been widely used to assess multi-organ damage for sepsis, especially in the ICU settings [2,3]. The SOFA score is a sum of scores for the brain, respiratory, cardiovascular, liver, kidney, and hemostasis functions (0–4 for each component). Infection is generally confirmed by the positive culture of the pathogens (growth of the pathogens from samples of body fluids or tissues) or tested for pathogen-specific antigen, antibody and genetic materials. As for the assessment of dysregulated host response, a variety of assays has been utilized. The routinely used serum markers for inflammation include C-reactive protein (CRP), procalcitonin (PCT), IL-6, TNFα and others [4]. For deeper understanding of sepsis, multi-omics technologies have been applied, including proteomics, genomics, transcriptomics, and metabolomics [5]. To address patient heterogeneity, sub-phenotyping approaches were investigated [6,7]. More recently, artificial intelligence (AI) was attempted for the improved management of sepsis [8]. Nevertheless, the only effective treatments for sepsis thus far remain to be antibiotic treatment and life support interventions. There is an urgent need to find promising therapeutic targets to reduce the mortality of sepsis.

According to the current understanding, the dysregulated host response encompasses several compartments of the immune system [9]. Excessive release of cytokines is caused by neutrophil degranulation (or neutrophil extracellular traps, NETs) [10,11] and prolonged activation of interferon signaling [12]. Apart from this hyper-inflammation, the so-called immuno-paralysis is also observed in sepsis [13]. This is reflected in lymphopenia [14], deficiency in dendritic cells [15], decreased expression of monocyte HLA-DR [16], and other aspects. Ideally, a side-by-side comparison of these immune compartments may point out the more promising targets for immune modulation in sepsis.

In our previous works, we have identified some of the most prominent marker genes in the peripheral blood of infectious and immunological conditions. For non-severe infection, type I interferon-stimulated genes (the ISGa gene set) and neutrophil degranulation-related genes (the NDrG gene set) were sufficient for the classification of viral and bacterial infections [17,18,19]. Activation of NDrG was also observed in severe viral infection [20,21,22], accompanied by the suppression of the HLA-D gene cluster (the HLAd gene set) [23]. Furthermore, prolonged activation of ISGa and GBPs (the GBP gene cluster for type II interferon signaling) was also observed in severe viral infection [23]. In addition, marker genes for T cells, B cells, NK cells and dendritic cells were confirmed in our previous single-cell RNA-Seq analysis [24].

In this work, we simultaneously evaluated five components of host immunity in sepsis with a composite gene panel of five gene sets, including NDrG, ISGa, GBPs, HLAd and LYMd (for lymphocytes and dendritic cells). The relevance of the gene sets with the pathogenesis of sepsis is proposed in Supplementary Figure S1. The evaluation was based on seven relevant public transcriptome datasets on peripheral blood using RNA-seq technology. We were interested in (1) whether these components were significantly different between sepsis patients and healthy controls, (2) whether these components were correlated with the hyper-inflammation phenotype, (3) whether the serial change in these components was correlated with the improved SOFA score during the treatment, (4) whether differential response to treatment could be reflected on these components and (5) whether the initial assessment at ICU admission was associated with the outcome. Throughout these analyses, it may become clear which components may be more promising for the purpose of prognosis and therapeutic targeting.

## 2. Materials and Methods

The transcriptome datasets analyzed in this work were downloaded from gene expression omnibus (GEO, https://www.ncbi.nlm.nih.gov/geo/, accessed on 1 February 2026). All these studies were performed with RNA-Seq (RNA sequencing) platforms. The tissue sources were peripheral blood. Five groups of marker genes were evaluated in this work. The NDrG gene set for neutrophil degranulation-related genes consisted of *S100A12*, *CD177*, *HP*, *ANXA3*, and *ARG1*. The ISGa gene set for type I interferon signaling consisted of *IFI27*, *RSAD2*, *IFI44L*, *ISG15* and *IFITM3*. The GBPs gene set for type II interferon signaling consisted of *GBP1*, *GBP2*, *GBP3*, *GBP4* and *GBP5*. The HLAd gene set for MHC class II consisted of *HLA-DRA*, *HLA-DRB1*, *HLA-DMA*, *HLA-DMB* and *HLA-DPA1*. The LYMd gene set for lymphocytes and dendritic cells consisted of *CD4*, *CD8A*, *CD79A*, *CD27*, *KLRF1* and *FCER1A*.

Seven relevant transcriptome datasets were analyzed in this work (Table 1). Each of the datasets was analyzed independently. One dataset GSE228541 was focused on the comparison between sepsis patients and healthy controls [25]. Another dataset GSE236892 was focused on the comparison between sepsis patients with hyper-inflammation or hypo-inflammation [26]. The third dataset GSE184039 was focused on the comparison of patients who had high (>250) or low (<100) CRP values after a major surgery [27]. In another dataset GSE131411, patients with septic shock in the ICU were evaluated at three time points, within 16 h of ICU admission, 48 h later and day 7 or discharge [28]. Comparison of patients with differential response to treatment (responders and non-responders) was conducted in two datasets, including the dataset GSE110487 for the comparison of the two groups at two time points (T1 for ICU admission and T2 for 48 h later) [29], and the dataset GSE216902 for the comparison of the two groups at day 1 and day 8 of ICU admission [30]. The last dataset GSE185263 was focused on the comparison of differential outcome for sepsis patients admitted to ICU [31].

For each transcriptome dataset, the expression values of the marker genes were extracted from the expression matrix. The raw counts were converted to normalized counts and log 2 transformed. The mean expression values of the component genes in each sample were used to represent the marker gene sets (the NDrG, ISGa, GBPs, HLAd and LYMd gene sets). Since healthy controls were not available in the majority of the datasets, no further normalization of the gene expression was performed.

The statistical calculations were performed in R (version 4.5.1, https://www.r-project.org/, accessed on 1 February 2026). For pairwise comparison of the same patients at two time points, the paired t test was used. For multi-group comparison, the Tukey HSD (honest significant difference) test was used. The principal component analysis (PCA) was also conducted in R. The plots were generated with the R packages ggplot2 (general plotting), gridExtra (arrangement of multi-graphs), and rgl (3D plotting). The 3D plot and PCA plot were generated in R studio (version 2026.01.0, https://posit.co/downloads/, accessed on 1 February 2026).

## 3. Results

Built upon our previous works, we evaluated several components of the host immunity in sepsis. The marker gene panel consisted of five gene sets, including NDrG for neutrophil degranulation, ISGa for type I interferon signaling, GBPs for type II interferon signaling, HLAd for MHC class II, and LYMd for lymphocytes and dendritic cells. Among these five gene sets, the NDrG, ISGa, GBPs and HLAd gene sets were developed from our previous works on COVID-19 and other conditions, while the LYMd gene set was newly developed here to reflect the deficiency of lymphocytes and dendritic cells in sepsis. The NDrG gene set consisted of *S100A12*, *CD177*, *HP*, *ANXA3*, and *ARG1*. The ISGa gene set consisted of *IFI27*, *RSAD2*, *IFI44L*, *ISG15* and *IFITM3*. The GBPs gene set consisted of *GBP1*, *GBP2*, *GBP3*, *GBP4* and *GBP5*. The HLAd gene set consisted of *HLA-DRA*, *HLA-DRB1*, *HLA-DMA*, *HLA-DMB* and *HLA-DPA1*. The LYMd gene set consisted of *CD4*, *CD8A*, *CD79A*, *CD27*, *KLRF1* and *FCER1A*. Using this composite marker gene panel, we evaluated immune dysregulation in seven datasets related to sepsis (Table 1). For each of the five gene sets, the mean expression of the component genes was used.

### 3.1. Dysregulation of the Marker Gene Sets in Sepsis

**Figure 1:**
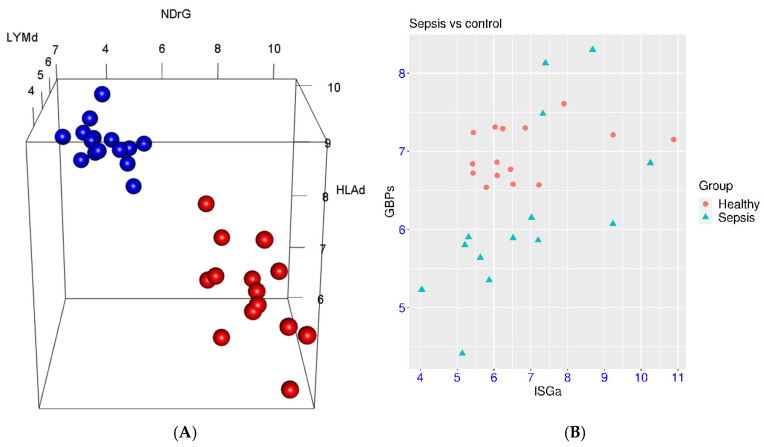
Expression patterns of marker gene sets in sepsis (GSE228541). (**A**), Left panel, distinctive 3D distribution of NDrG/HLAd/LYMd expression in the sepsis patients (red) and healthy controls (blue). (**B**), Right panel, expression of marker gene sets for interferon signaling (ISGa and GBPs) in sepsis and healthy controls.

First, the marker gene panel was examined in a dataset with both sepsis patients and healthy controls (GSE228541). In this small cohort, the sepsis patients and healthy controls could be clearly separated by the NDrG, HLAd and LYMd gene sets (Figure 1A). The *p*-values for the two-group comparison were 8.23 × 10^−14^, 5.70 × 10^−9^, and 2.33 × 10^−9^, respectively. The mean differences between the two groups were 5.0, −1.75, and −2.18, respectively. Thus, there were simultaneous activations of NDrG and suppressions of HLAd and LYMd in sepsis. As for the marker genes for interferon signaling, no activation of ISGa was observed in this small cohort, while suppression of GBPs was observed in 10 of the 14 sepsis patients (Figure 1B).

### 3.2. Association with Hyper-Inflammation

**Figure 2:**
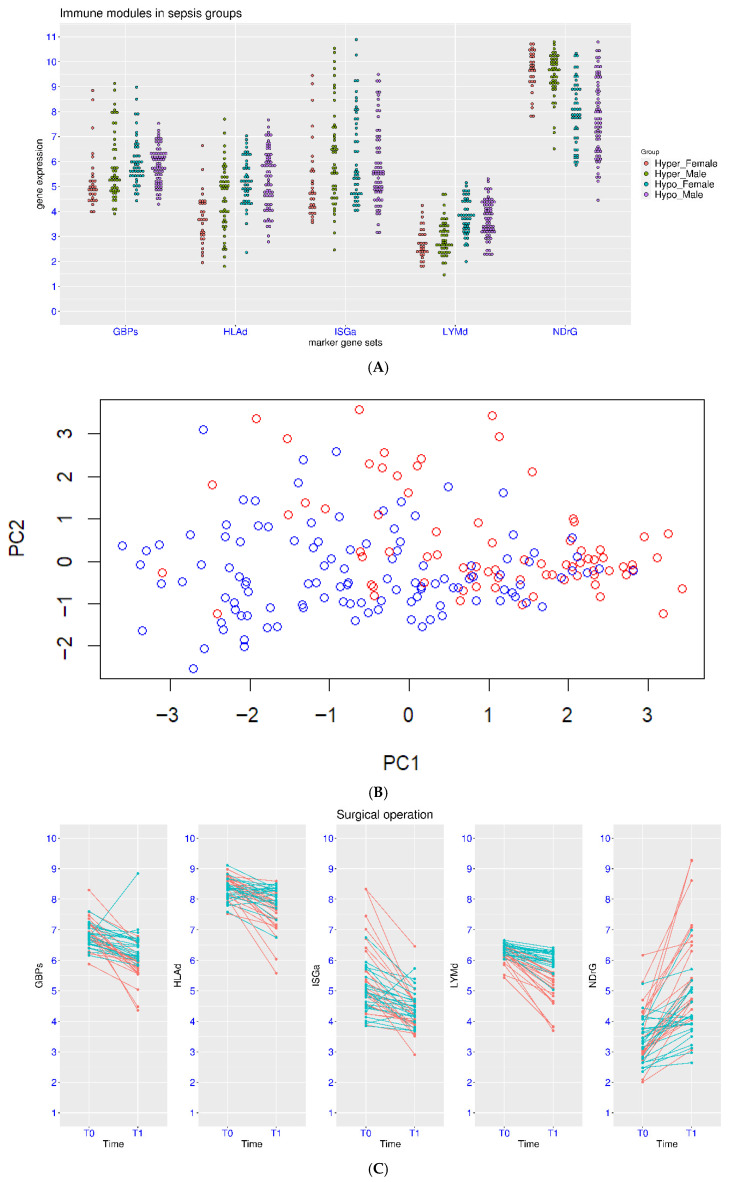
Expression patterns of the marker gene sets in patients with different inflammation status. (**A**), top panel, expression patterns in sepsis patients with hyper-inflammation or hypo-inflammation (GSE236892). (**B**), middle panel, distribution of the samples on the PCA map (GSE236892, red for hyper-inflammation and blue for hypo-inflammation). PC1 was the first principle component, ans PC2 was the second principle component. (**C**), bottom panel, change in gene expression before and after a major surgery (T0 and T1, respectively). The patients were grouped based on high (red) or low (cyan) CRP levels (GSE184039).

Hyper-inflammation is associated with unfavorable outcome for sepsis patients. In the dataset GSE236892, sepsis patients were divided into hyper-inflammation and hypo-inflammation groups based on clinical features and serum biomarkers. Due to the relatively large sample size in this dataset, patients were further divided into male and female subgroups. It was evident that simultaneous activation of NDrG and suppression of HLAd and LYMd was observed in the hyper-inflammation group compared to the hypo-inflammation group (Figure 2A). For NDrG, the *p*-value from group comparison was 6.0 × 10^−7^ and <1.0 × 10^−7^ for females and males, respectively. For HLAd, the *p*-value from group comparison was 6.0 × 10^−7^ and 6.0 × 10^−3^ for females and males, respectively. For LYMd, the *p*-value from group comparison was <1.0 × 10^−7^ and 6.0 × 10^−7^ for females and males, respectively. Therefore, higher NDrG values and lower HLAd and LYMd values may be associated with unfavorable outcomes for sepsis patients (both males and females). As for the marker gene sets for interferon signaling, statistical significance was only reached for GBPs in the female groups (*p* = 0.013). Additionally, the correlation between age and expression values of the marker gene sets was analyzed for this dataset, and no correlation was observed between age and expression values of any of the five marker gene sets. Principal component analysis (PCA) was also conducted on this dataset (Figure 2B). The NDrG, HLAd and LYMd gene sets contributed more to PC1, while the ISGa and GBPs gene sets contributed more to PC2. Patients with higher PC1 or PC2 values tended to have hyper-inflammation, while samples in the lower left corner of the graph were mostly patients with hypo-inflammation.

Another simple evaluation of hyper-inflammation is the high CRP values, which could be caused by major surgery and may raise the risk of sepsis. In one such study, patients were grouped based on the CRP values after a major surgery (GSE184039). None of the marker gene sets showed statistical significance between the two groups before the surgery (Figure 2B). At the second time point, the *p*-values from the group comparison were 2.04 × 10^−4^, 8.43 × 10^−4^, and <1.0 × 10^−7^, for NDrG, HLAd and LYMd, respectively. Again, hyper-inflammation was accompanied by simultaneous activation of NDrG and suppression of HLAd and LYMd. In addition, LYMd displayed relatively stronger association with the CRP levels. As for the marker gene sets for interferon signaling, statistical significance was only reached for GBPs for the group comparison (*p* = 6.0 × 10^−5^). Thus, suppression of GBPs may also be associated with hyper-inflammation.

### 3.3. Application to the Monitoring of Treatment Response

**Figure 3:**
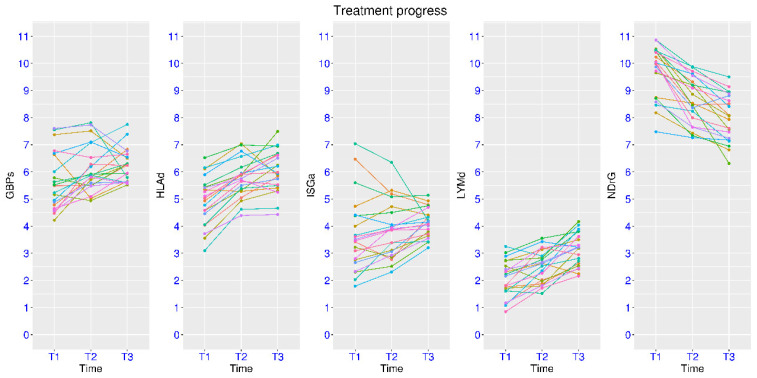
Reversal of gene dysregulation upon treatment (GSE131411). Expression of the marker gene sets at three time points of the treatment process for sepsis patients. T1 for within 16 h of ICU admission, T2 for 48 h later and T3 for day 7 or discharge.

Since this marker gene panel is associated with hyper-inflammation, it may also be applicable to the monitoring of treatment response. In a related dataset GSE131411, patients with septic shock were evaluated at three time points—T1 for within 16 h of ICU admission, T2 for 48 h after ICU admission, and T3 for day 7 or before discharge. These patients had improved SOFA scores from T1 to T3. Accordingly, a clear trend of decreased NDrG and increased HLAd and LYMd was observed from T1 to T3 (Figure 3). From paired tests, the mean differences were −1.70, +1.06, and +1.08, and the *p*-values were 4.98 × 10^−9^, 4.02 × 10^−7^, and 1.07 × 10^−7^, respectively. Considering the dysregulation pattern of these three marker gene sets presented earlier, it was a clear reversal of gene dysregulation for all three marker gene sets. As for the marker gene sets for interferon signaling, no clear trend was observed for GBPs or ISGa from T1 to T3. Changes in both directions were observed. Unfortunately, healthy controls were not available in this dataset. However, this may still be the reversal of gene dysregulation, since both activation and suppression may be present for different patients as shown in a later dataset with healthy controls included.

**Figure 4:**
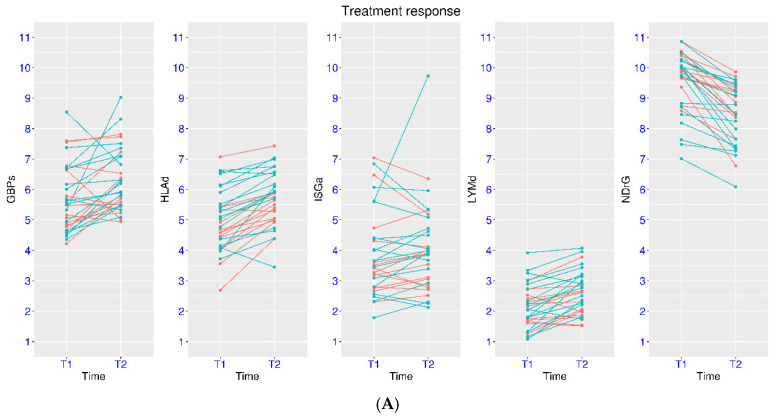

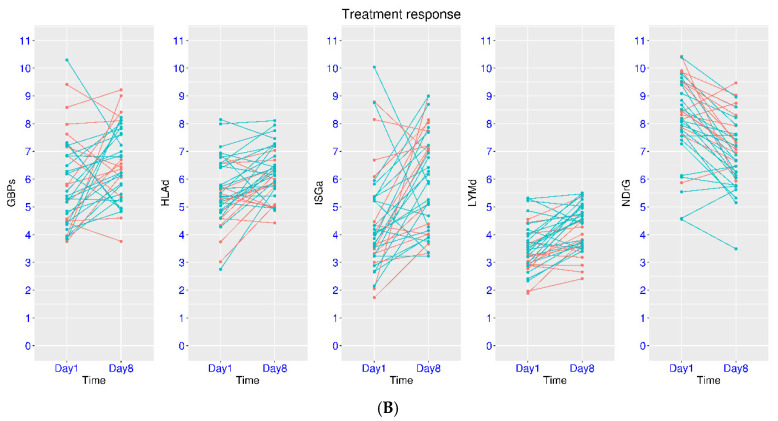
Expression pattern of the marker gene sets in sepsis patients with different treatment response. (**A**), upper panel, expression at ICU admission (T1) and 48 h later (T2) (GSE110487). (**B**), lower panel, expression at ICU admission (day 1) and day 8 (GSE216902). In both panels, cyan color was for responders and red color was for non-responders.

Due to patient heterogeneity, some patients may respond better to the treatment than others do. In one such study (GSE110487), patients with septic shock were evaluated at ICU admission (T1) and 48 h later (T2). Patients were classified as responders and non-responders based on the SOFA scores at the two time points. In fact, significant changes were observed for NDrG, HLAd and LYMd for both groups using paired tests (Figure 4A). For the responder group, the *p*-values were 2.20 × 10^−5^, 3.97 × 10^−4^, and 1.25 × 10^−4^, and the mean differences were −1.04, +0.63, and +0.64, respectively. For the non-responder group, the *p*-values were 5.81 × 10^−5^, 3.94 × 10^−5^, and 0.028, and the mean differences were −1.08, +0.80, and +0.28, respectively. From unpaired multi-group tests, the most significant difference for these three marker gene sets was between the responders at T2 and non-responders at T1. The *p*-values were 1.04 × 10^−4^, 3.60 × 10^−3^, and 2.96 × 10^−3^, and the mean differences were −1.63, +1.22, and +0.93, respectively. Additionally, subtle differences could also be observed between the two groups. For example, the five patients with the lowest NDrG values at T1 belonged to the responder group. As for the marker gene sets for interferon signaling, statistical significance was only reached for GBPs in the responder group, with *p*-value of 0.012 and mean difference of +0.77 between the two time points. It shall be noted that this kind of test may not be appropriate if changes in both directions are expected for different patients.

In another study on treatment response (GSE216902), sepsis patients were evaluated at day 1 and day 8 of ICU admission (Figure 4B). Paired tests were conducted. For the responder group, the *p*-values were 1.32 × 10^−5^, 5.73 × 10^−3^, and 3.41 × 10^−4^, and the mean differences were −1.26, +0.71, and +0.78, respectively for NDrG, HLAd and LYMd. For the non-responder group, the *p*-values were 0.015, 0.059, and 0.0092, and the mean differences were −1.06, +0.58, and +0.56, respectively. Therefore, the change was more pronounced in the responder group. From unpaired multi-group tests, the most significant difference for these three marker gene sets was between the responders at day 8 and non-responders at day 1. The *p*-values were 8.39 × 10^−5^, 1.68 × 10^−3^, and 1.54 × 10^−4^, and the mean differences were −2.10, +1.34, and +1.23, respectively. Other subtle differences were also observed. A few patients with the lowest NDrG values or highest HLAd or LYMd values at day 1 belonged to the responder group. As for the marker gene sets for interferon signaling, significant increase in ISGa was observed for both groups from day 1 to day 8. However, as indicated earlier, this kind of test may not be appropriate if changes in both directions are expected.

### 3.4. Association with the Outcome of ICU Patients

**Figure 5:**
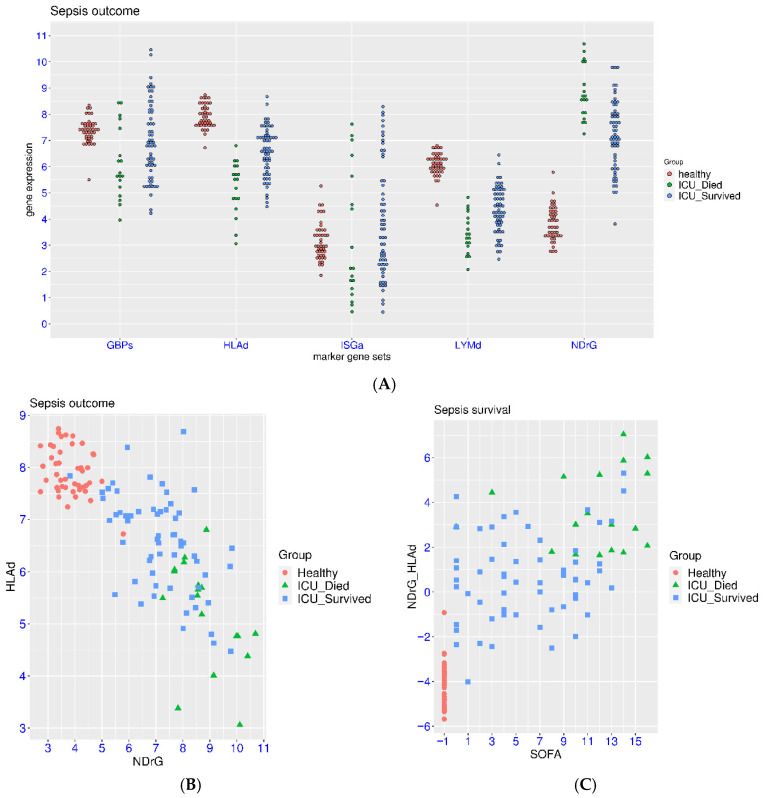
Correlation of the expression of the marker gene sets and SOFA scores with the outcome of the sepsis patients (GSE185263). (**A**), expression patterns in healthy controls and ICU patients grouped by survival. (**B**), distribution of sepsis patients and healthy controls on a 2D map of NDrG and HLAd values. (**C**), distribution of sepsis patients and healthy controls on a 2D map of SOFA score and (NDrG-HLAd) values. For clarity, the SOFA scores for the healthy controls were arbitrarily assigned to −1.

In addition to the association with hyper-inflammation and treatment response, we also examined whether the initial measurement of this gene panel was associated with the outcome of the sepsis patients. In a related dataset (GSE185263), both healthy controls and sepsis patient admitted to ICU were included, giving a clear indication of deviation from normal range for these marker gene sets. The patients were grouped based on survival. Significant activation of NDrG and suppression of HLAd and LYMd were observed in sepsis patients compared to healthy controls (Figure 5A). The *p*-values were <1.0 × 10^−7^ for all three parameters in either sepsis group compared with the healthy controls. Difference between the two patient groups was observed. The *p*-values were 6.09 × 10^−4^, 1.20 × 10^−6^, and 2.62 × 10^−3^, and the mean differences were −1.71, 1.39, and 0.92, respectively. The dysregulation was uni-directional for these three marker gene sets. As for the marker gene sets for interferon signaling, both activation and suppression were observed. However, significant suppression of GBPs was observed for the non-survival or fatal group. When compared with the healthy controls, the *p*-value was 5.06 × 10^−3^ and the mean difference was 1.30. Overall, patients with NDrG/HLAd/HLAd values closer to the normal range in healthy controls had much better chance of survival (Figure 5B). For example, 32 patients with the lowest NDrG values all survived, 26 patients with the highest HLAd values all survived, and 19 patients with the highest LYMd values also survived. A simple combination of NDrG and HLAd ((NDrG-HLAd) < 1.50) could identify 45 of the 60 survivors (75%) with zero false positives (Figure 5C). On the other hand, the correlation between the outcome and the marker gene sets for interferon signaling was not as clear especially for ISGa. As a comparison, 36 of the 60 survivors (60%) could be identified by SOFA < 8 with 2 non-survivors misidentified (Figure 5C). The combination of either (NDrG-HLAd) < 1.50 or SOFA < 8 could identify 54 of the 60 survivors (90%) with only 2 false positives. Correlation analysis was conducted between the SOFA scores and the expression values of the marker gene sets. Only moderate correlation between NDrG and SOFA (r = 0.508) and weak correlation between HLAd or LYMd and SOFA (r = −0.294 and −0.255) were observed. No correlation was observed between GBPs or ISGa and SOFA (r = −0.096 and −0.088).

## 4. Discussion

Pinpointing the major source of the health risk in sepsis is crucial for the development of therapeutic interventions targeting the specific risks [7,32,33]. By comparing five components of the host immunity, it seemed that highly elevated NDrG expression and suppressed HLAd expression were more correlated with the outcome than the other three factors. The simple combination of (NDrG-HLAd) was sufficient to identify 75% of the survivors in the dataset described above. It suggests that moderate elevation of NDrG expression and suppression of HLAd expression are not fatal for sepsis patients admitted to ICU. The exact cutoff value needs to be decided with the specific platform for gene expression assay. The subtraction model also means that no reference genes may be needed which could reduce the number of genes for the assay. Further simplification of the model may be tested by selecting fewer genes based on assays of larger cohorts in future trials. Although the relatively higher LYMd expression was also associated with favorable outcome, those patients also had relatively lower (NDrG-HLAd) values. Therefore, LYMd could be excluded from the simplified model.

Integration of SOFA score with other complimentary parameters has been attempted in previous investigations with modest improvement [34]. Based on the analysis here, the change in (NDrG-HLAd) values was correlated with the change in SOFA scores in the serial monitoring during the treatment process. The prognosis performance was better for the (NDrG-HLAd) value than the SOFA score which is the gold standard in the ICU settings. In the meantime, these two parameters were complimentary to each other when used in the identification of survivors in ICU. The SOFA scores could capture the degree of multi-organ damage, while the (NDrG-HLAd) values could capture the degree of immune dysregulation. Less deviation from the normal range for either multi-organ damage or immune dysregulation may be sufficient for the favorable outcome. Although the idea of immune modulation for sepsis is not accepted by some researchers [35], it still deserves attention [36]. The strong correlation of the (NDrG-HLAd) values with the outcome suggests that immune modulation specifically targeting NDrG and HLAd expression may be a promising direction.

The roles of ISGa and GBPs in sepsis are more complex. First, both activation and suppression could be observed according to the dataset GSE185263. Some of the patients with normal ISGa or GBPs expression did not have favorable outcome. Interestingly, the 11 patients with GBPs > 8.5 all survived, suggesting that activation of GBPs expression is at least not detrimental. The incorporation of the GBP value could identify 2 more survivors (56 of the 60 survivors in total) with no more false positives. It may suggest that proper activation of type II interferon signaling may be beneficial to some of the sepsis patients. The correlation of ISGa expression with the outcome was even less clear. Nevertheless, these two parameters may still deserve further investigation in future studies. For example, stratification of the patients into viral or bacterial infection may render help with the interpretation of the data. The time from the initiation of viral infection is also crucial information for the understanding of dysregulation in ISGa and GBPs.

The work is mainly limited by the availability of high-quality transcriptome data. Healthy controls were included in only two of the seven RNA-Seq datasets examined here. It would be impossible to assess the deviation from normal range without healthy controls. It would also be desirable to have at least one more dataset like GSE185263 for independent observation. Unfortunately, we do not have the resources to conduct this kind of validation, which is a major limitation of the work. We welcome the clinical research community to evaluate this newly proposed assay. Nevertheless, parameter fitting was not attempted in this work in order to avoid over-fitting and assist in clearer interpretation of the model and potentially higher reproducibility.

Due to the co-existence of hyper-inflammation and immune-suppression in sepsis, it is unlikely that non-specific suppression or activation of the immune system will improve the current situation [37,38,39,40]. The ultimate goal of this work is to find specific targets for immune modulation. From this work, it seems that neutrophil degranulation and MHC class II could be promising targets [41,42]. If this is confirmed repeatedly in future investigations with large cohorts, the next logical step would be testing specific immune modulation strategy in animal models and clinical trials.

## 5. Conclusions

In this work, five components of the host immunity were evaluated in sepsis. Activation of NDrG and suppression of HLAd and LYMd were consistently observed in sepsis. Hyper-inflammation was correlated with higher NDrG expression and lower HLAd and LYMd expression. The reversal of gene dysregulation was observed during the treatment process especially for the responders. The initial (NDrG-HLAd) value at ICU admission could identify 75% of the survivors with 0% false positive rate, while the combination of (NDrG-HLAd) value with the SOFA score could identify 90% of the survivors with 3.5% false positive rate. Overall, the NDrG and HLAd components were the more promising targets for immune modulation in sepsis. Future works are warranted to further examine the value of these immune components in sepsis.

## Figures and Tables

**Table 1:** Transcriptome datasets for the evaluation of immune dysregulation in sepsis.

Accession ID	Condition	Sample Numbers	Age	Ref.
GSE228541	Sepsis due to community-acquired pneumonia	15 controls, 14 sepsis patients, 11 intubated	40–59, controls 67–84, patients	[25]
GSE236892	Sepsis	113, hypo group, 76, hyper group, 86% intubated	Hypo, 65 (15) Hyper, 66 (15) Mean (SD)	[26]
GSE184039	Patients with high or low CRP after major surgery	21 with high CRP, 25 with low CRP, 2 time points	43–87, high CRP 26–80, low CRP	[27]
GSE131411	Patients with septic shock in ICU	21 patients, 3 time points	67.5 (19.2) mean (SD)	[28]
GSE110487	Patients with septic shock in ICU	14 non-responders, 17 responders, 2 time points	NonR, 68.6 (20.7) Resp, 62.8 (18.4) Mean (SD)	[29]
GSE216902	Sepsis patients in ICU	14 non-responders, 23 responders, 2 time points	NonR, 83 (79–88) Resp, 82 (79–86) Mean (range)	[30]
GSE185263	Sepsis patients in ICU	60 survived, 18 died, 44 healthy controls	Sepsis: 61.7 (1.7) mean (SD)	[31]
Total		631 samples		

## Data Availability

All of the datasets analyzed in the current work are publicly available. The transcriptome datasets are available at GEO (gene expression omnibus, https://www.ncbi.nlm.nih.gov/geo/, accessed on 1 February 2026). For more details, please refer to the Section 2 and Table 1.

## Supplementary Materials

The following supporting information can be downloaded at https://www.mdpi.com/article/10.3390/biomedicines14030617/s1, Figure S1: A proposed mechanism for the roles of the five immune components in the pathogenesis of sepsis.

## References

[1] Singer M., Deutschman C.S., Seymour C.W., Shankar-Hari M., Annane D., Bauer M., Bellomo R., Bernard G.R., Chiche J.D., Coopersmith C.M. (2016). The Third International Consensus Definitions for Sepsis and Septic Shock (Sepsis-3). JAMA.

[2] Talkar M.A., Meena D.S., Kumar D., Bohra G.K., Tak V., Rohila A.K., Sharma A., Garg M.K. (2025). The predictive performance of NEWS, MEWS, SOFA and SAPS II in outcomes of bacteremic and non-bacteremic sepsis. BMC Infect. Dis..

[3] Ranzani O.T., Singer M., Salluh J.I.F., Shankar-Hari M., Pilcher D., Berger-Estilita J., Coopersmith C.M., Juffermans N.P., Laffey J., Reinikainen M. (2025). Development and Validation of the Sequential Organ Failure Assessment (SOFA)-2 Score. JAMA.

[4] Bourika V., Rekoumi E.A., Giamarellos-Bourboulis E.J. (2025). Biomarkers to guide sepsis management. Ann. Intensive Care.

[5] Jin X., Shen H., Zhou P., Yang J., Yang S., Ni H., Yu Y., Zhang Z. (2025). Research Progress on Sepsis Diagnosis and Monitoring Based on Omics Technologies: A Review. Diagnostics.

[6] Kolodyazhna A., Wiersinga W.J., van der Poll T. (2025). Aiming for precision: Personalized medicine through sepsis subtyping. Burn. Trauma.

[7] Van Nynatten L.R., Bokhary D., Wong M.Y.S., Wang J., Fero H., McChesney C., Fiorini K., Blake L., Fraser D.D., Slessarev M. (2025). Predictive enrichment using biomarkers in studies of critically-ill patients with sepsis: A systematic review. Crit. Care.

[8] Hernandez B., Ming D.K., Rawson T.M., Bolton W., Wilson R., Vasikasin V., Daniels J., Rodriguez-Manzano J., Davies F.J., Georgiou P. (2025). Advances in diagnosis and prognosis of bacteraemia, bloodstream infection, and sepsis using machine learning: A comprehensive living literature review. Artif. Intell. Med..

[9] Valsamaki A., Vazgiourakis V., Mantzarlis K., Manoulakas E., Makris D. (2025). Immune Dysregulation in Sepsis. A Narrative Review for the Clinicians. Biomedicines.

[10] Zhang J., Shao Y., Wu J., Zhang J., Xiong X., Mao J., Wei Y., Miao C., Zhang H. (2025). Dysregulation of neutrophil in sepsis: Recent insights and advances. Cell Commun. Signal..

[11] Vella R., Panci D., Carini F., Malta G., Vieni S., David S., Albano G.D., Puntarello M., Zerbo S., Argo A. (2025). Cytokines in sepsis: A critical review of the literature on systemic inflammation and multiple organ dysfunction. Front. Immunol..

[12] Gürtler L.G., Schramm W., Seitz R. (2025). Viral sepsis—Pathophysiology and disease manifestation. Infection.

[13] Saavedra-Torres J.S., Pinzon-Fernandez M.V., Nati-Castillo H.A., Cadena Correa V., Lopez Molina L.C., Gaitan J.E., Tenorio-Castro D., Lucero Guanga D.A., Arias-Intriago M., Tello-De-la-Torre A. (2025). Immunodynamic Disruption in Sepsis: Mechanisms and Strategies for Personalized Immunomodulation. Biomedicines.

[14] Nedel W., Henrique L.R., Portela L.V. (2025). Why should lymphocytes immune profile matter in sepsis?. World J. Crit. Care Med..

[15] Tong S., Zhang T., Chen N., Liu J.P., Wei S.T., Hua T.Z., Duan Y., Sun B., Dong N., Wu Y. (2026). Tripartite motif 13 orchestrates endoplasmic reticulum-associated degradation and endoplasmic reticulum-phagy to modulate dendritic cell-mediated immune responses in sepsis. Burn. Trauma.

[16] Monneret G., Lafon T., Gossez M., Evrard B., Bodinier M., Rimmele T., Argaud L., Cour M., Friggeri A., Lepape A. (2025). Monocyte HLA-DR expression in septic shock patients: Insights from a 20-year real-world cohort of 1023 cases. Intensive Care Med..

[17] Song F., Qian Y., Peng X., Li X., Xing P., Ye D., Lei H. (2017). The frontline of immune response in peripheral blood. PLoS ONE.

[18] Lei H., Xu X., Wang C., Xue D., Wang C., Chen J. (2021). A host-based two-gene model for the identification of bacterial infection in general clinical settings. Int. J. Infect. Dis..

[19] Lei H. (2024). Quantitative and Longitudinal Assessment of Systemic Innate Immunity in Health and Disease Using a 2D Gene Model. Biomedicines.

[20] Lei H. (2021). A single transcript for the prognosis of disease severity in COVID-19 patients. Sci. Rep..

[21] Lei H. (2023). A two-gene marker for the two-tiered innate immune response in COVID-19 patients. PLoS ONE.

[22] Lei H. (2024). Hypoxia and Activation of Neutrophil Degranulation-Related Genes in the Peripheral Blood of COVID-19 Patients. Viruses.

[23] Lei H. (2025). Distinctive Temporal Profiles of Interferon-Stimulated Genes in Natural Infection, Viral Challenge, and Vaccination. Viruses.

[24] Lei H., Wang C., Wang Y., Wang C. (2021). Single-cell RNA-Seq revealed profound immune alteration in the peripheral blood of patients with bacterial infection. Int. J. Infect. Dis..

[25] Oda S., Matsumoto H., Togami Y., Yoshimura J., Ito H., Onishi S., Muratsu A., Mitsuyama Y., Okuzaki D., Ogura H. (2025). mRNA-miRNA integration analysis of T-cell exhaustion in sepsis from community-acquired pneumonia. Acute Med. Surg..

[26] Neyton L.P.A., Sinha P., Sarma A., Mick E., Kalantar K., Chen S., Wu N., Delucchi K., Zhuo H., Bos L.D.J. (2024). Host and Microbe Blood Metagenomics Reveals Key Pathways Characterizing Critical Illness Phenotypes. Am. J. Respir. Crit. Care Med..

[27] Bain C.R., Myles P.S., Taylor R., Trahair H., Lee Y.P., Croft L., Peyton P.J., Painter T., Chan M.T.V., Wallace S. (2022). Methylomic and transcriptomic characterization of postoperative systemic inflammatory dysregulation. Transl. Res..

[28] Braga D., Barcella M., Herpain A., Aletti F., Kistler E.B., Bollen Pinto B., Bendjelid K., Barlassina C. (2019). A longitudinal study highlights shared aspects of the transcriptomic response to cardiogenic and septic shock. Crit. Care.

[29] Barcella M., Bollen Pinto B., Braga D., D’Avila F., Tagliaferri F., Cazalis M.A., Monneret G., Herpain A., Bendjelid K., Barlassina C. (2018). Identification of a transcriptome profile associated with improvement of organ function in septic shock patients after early supportive therapy. Crit. Care.

[30] Chen I.C., Chen H.H., Jiang Y.H., Hsiao T.H., Ko T.M., Chao W.C. (2023). Whole transcriptome analysis to explore the impaired immunological features in critically ill elderly patients with sepsis. J. Transl. Med..

[31] Baghela A., Pena O.M., Lee A.H., Baquir B., Falsafi R., An A., Farmer S.W., Hurlburt A., Mondragon-Cardona A., Rivera J.D. (2022). Predicting sepsis severity at first clinical presentation: The role of endotypes and mechanistic signatures. EBioMedicine.

[32] Qin C., Ma D., Pang L., Hu M., Lin S., Zhou Z., Xu X., Ji C. (2025). Prognostic factors of sepsis: A systematic review and meta-analysis. BMC Infect. Dis..

[33] Xu Z., Zhang J., Li Z., Wu H., Xu H., Guo Y., Li Y. (2025). Organ-targeted biomarkers of sepsis: A systematic review reveals the value of inflammation and lipid metabolic dysregulation. Pharmacol. Res..

[34] Zhu X., Li W., Lv Z., Pan X. (2025). Integration of metabolic parameters with SOFA score for mortality risk assessment in elderly sepsis: A derivation study. BMC Infect. Dis..

[35] Feng Z., Wang L., Yang J., Li T., Liao X., Kang Y., Xiao F., Zhang W. (2025). Sepsis: The evolution of molecular pathogenesis concepts and clinical management. MedComm.

[36] Arapis A., Panagiotopoulos D., Giamarellos-Bourboulis E.J. (2025). Recent advances of precision immunotherapy in sepsis. Burn. Trauma.

[37] Vincent J.L. (2025). The 15th Anniversary of Life-Sepsis Trials. Life.

[38] Vernay E., Cerrato E., Santinon F., Monard C., Perez P., Allantaz F., Lukaszewicz A.C., Llitjos J.F. (2025). Considering local immunity for innovative immunomodulatory approaches: Pulmonary sepsis as a use case. Front. Immunol..

[39] Gao Q.F., Teng Y.J., Zhu L., Zhang W., Li Z.Z. (2025). The immunosuppressive mechanisms induced by sepsis and the corresponding treatment strategies. Front. Immunol..

[40] You W.B. (2025). Roles of cytokine storm in sepsis progression: Biomarkers, and emerging therapeutic strategies. Front. Immunol..

[41] Pignataro G., Gemma S., Petrucci M., Barone F., Piccioni A., Franceschi F., Candelli M. (2025). Unraveling NETs in Sepsis: From Cellular Mechanisms to Clinical Relevance. Int. J. Mol. Sci..

[42] Snow T.A.C., Villa A., Cesar A., Ryckaert F., Saleem N., Smyth D., Pan H., Flint J., Brealey D., Singer M. (2025). Challenges of monocyte HLA-DR targeted immunomodulation in sepsis-a prospective observational cohort study. Front. Immunol..

