# Supplementary material for: Evaluation of Immune Dysregulation in Sepsis with a Composite Marker Gene Panel
Source: Biomedicines. 2026 Mar 10;14(3):617. doi: 10.3390/biomedicines14030617 (PMC13023613; doi:10.3390/biomedicines14030617)
Supplement: Supplementary file 1 [file biomedicines-14-00617-s001.zip › biomedicines-4180524-supplementary.pdf]

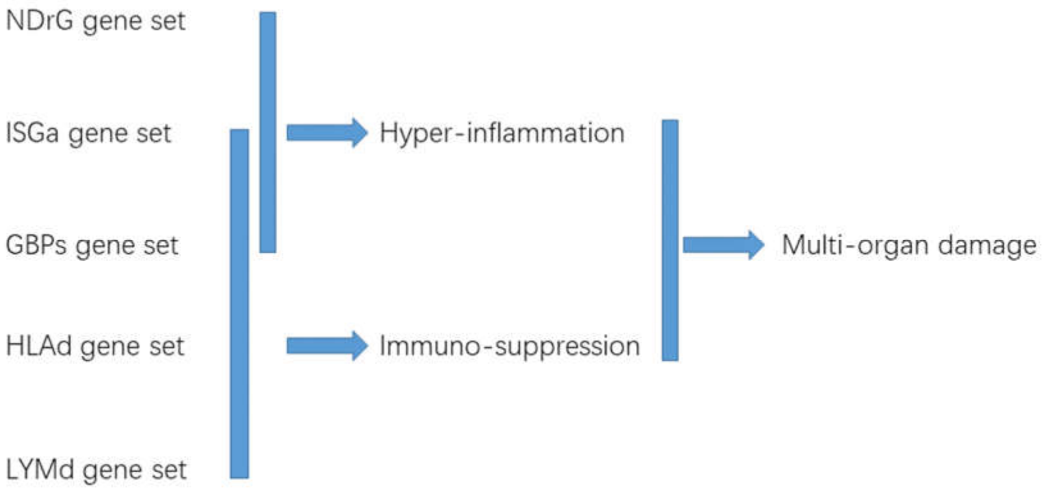

Supplementary Figure S1. A proposed mechanism for the roles of the five immune components in the pathogenesis of sepsis.
